# TRIM26 positively affects hepatitis B virus replication by inhibiting proteasome-dependent degradation of viral core protein

**DOI:** 10.1038/s41598-023-40688-3

**Published:** 2023-08-21

**Authors:** Yuki Nakaya, Tsutomu Nishizawa, Hironori Nishitsuji, Hiromi Morita, Tomoko Yamagata, Daichi Onomura, Kazumoto Murata

**Affiliations:** 1 Division of Virology, Department of Infection and Immunity, Jichi Medical University, Shimotsuke, 329-0498 Japan; 2 Department of Virology and Parasitology, School of Medicine, Fujita Health University, Toyoake, 470-1192 Japan

**Keywords:** Hepatitis B virus, Hepatitis B

## Abstract

Chronic hepatitis B virus (HBV) infection is a major medical concern worldwide. Current treatments for HBV infection effectively inhibit virus replication; however, these treatments cannot cure HBV and novel treatment-strategies should be necessary. In this study, we identified tripartite motif-containing protein 26 (TRIM26) could be a supportive factor for HBV replication. Small interfering RNA-mediated TRIM26 knockdown (KD) modestly attenuated HBV replication in human hepatocytes. Endogenous TRIM26 physically interacted with HBV core protein (HBc), but not polymerase and HBx, through the TRIM26 SPRY domain. Unexpectedly, TRIM26 inhibited HBc ubiquitination even though TRIM26 is an E3 ligase. HBc was degraded by TRIM26 KD in Huh-7 cells, whereas the reduction was restored by a proteasome inhibitor. RING domain-deleted TRIM26 mutant (TRIM26ΔR), a dominant negative form of TRIM26, sequestered TRIM26 from HBc, resulting in promoting HBc degradation. Taking together, this study demonstrated that HBV utilizes TRIM26 to avoid the proteasome-dependent HBc degradation. The interaction between TRIM26 and HBc might be a novel therapeutic target against HBV infection.

## Introduction

More than 250 million people worldwide suffer from chronic hepatitis B virus (HBV) infection^1^. Though pegylated-interferon (IFN) and nucleos(t)ide analogs (NUC) are currently used to treat chronic HBV infection for controlling virus replication, patients are required for life-long treatment because of the highly stable forms of the HBV genome, such as covalently closed circular DNA (cccDNA) and integrated HBV DNA^2,3^. Developing genome-editing technologies represented by CRISPR/Cas9 and TALEN systems might effectively eradicate the HBV genome; however, their safety and efficacy need to be fully assessed before clinical trials^4–6^.

Understanding host-virus interactions is important for establishing a treatment for virus infection. The host employs various molecules to restrict viral replication, while viruses interrupt host restriction by modulating molecular interactions^7–11^. HBV has a 3.2 kb genome DNA that produces four different mRNAs encoding surface (HBs), core (HBc), precore (HBe), and x (HBx) antigens, and polymerase (pol)^12^. In addition to the basic functions of virus replication, these proteins also counteract host defenses. For instance, HBs directly binds to bone marrow stromal cell antigen 2 (BST-2) to interrupt its dimerization, thus attenuating the anti-HBV activity of BST-2^13^. In another case, HBe downregulates Toll/IL-1 receptor (TIR)-mediated signaling by inhibiting TIR dimerization to evade host innate immunity^14^. However, the host-HBV interaction is not fully understood because of the lack of full knowledge of the HBV life cycle.

The tripartite motif (TRIM) family comprises many proteins with E3 ubiquitin ligase activity. TRIM family proteins have a common N-terminal structure composed of a RING finger domain, one or two B-box zinc finger domains, and a coiled-coil domain, while the C-terminal structures vary^15^. TRIM-containing protein 26 (TRIM26) has been identified as a supportive factor for the replication of Sendai virus, vesicular stomatitis virus (VSV), and herpes simplex virus 2 (HSV-2) ^16,17^. These viruses induce TRIM26 translocation from the cytoplasm to the nucleus, followed by TRIM26-dependent ubiquitination of nuclear, but not cytoplasmic, interferon regulatory factor 3 (IRF3) to promote its proteasome-dependent degradation, which attenuates type I and III IFN responses against viral infections^16,17^. On the other hand, TRIM26 helps hepatitis C virus (HCV) replication through the enhancement of the interaction between NS5A and NS5B by ubiquitinating NS5B without involvement of any IFN response^18^. Thus, TRIM26 potentially has supportive roles in the replication cycles of various viruses.

In the previous study, knockdown (KD) of TRIM26 attenuates HBV replication, although its molecular function remains veiled^19^. In this study, we focused on the molecular function of TRIM26 in HBV replication. We identified that TRIM26 depletion attenuated HBV replication in an IFN-independent manner. Mechanistically, TRIM26 interacts with HBc and inhibits its ubiquitination to prevent HBc from proteasome-dependent degradation. Thus, HBV uses TRIM26 for efficient replication.

## Results

### TRIM26 is required for efficient HBV replication in the infected cells

**Figure 1:**
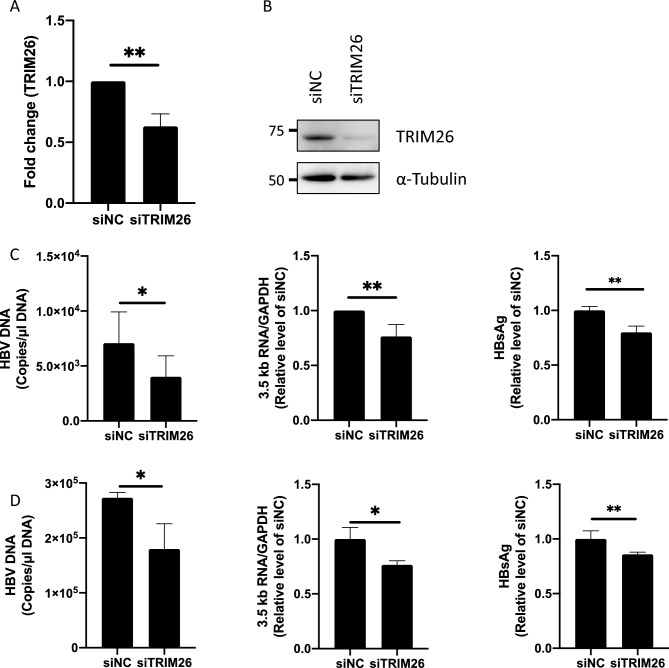
TRIM26 KD impairs HBV replication. (**A**) HepG2-NTCP cells were transfected with siNC or siTRIM26 and TRIM26 expression levels were analyzed by qPCR and western blotting. TRIM26 expression level was normalized by that of GAPDH and shown as the means ± standard deviations of three independent experiments. The full-length images are shown in Fig. S6. (**B**) TRIM26 protein levels were analyzed by western blotting. (**C**, **D**) HepG2-NTCP cells and PXB-cells were inoculated with HBV at the multiplicity of infection of 100 and 500 genome equivalents per cell (GEq/cell), respectively, and transfected with siNC or siTRIM26 at 4 days post inoculation (dpi). The culture medium was replaced with the fresh medium at 6 dpi, then the culture supernatants and cell lysates were harvested at 8 dpi to analyze HBV DNA, HBs (supernatants), and 3.5 kb RNA (lysates) levels. Values are the mean ± standard deviations of three independent experiments for HepG2-NTCP cells or a triplicate experiment for PXB-cells. All the significances were determined by student’s *t*-test and shown using asterisks (*P < 0.05, **P < 0.01).

To validate the previous finding that TRIM26 is required for the replication of the HBV reporter ^19^, we confirmed whether siRNA-mediated TRIM26 KD inhibits HBV replication in hepatocytes. Before the infection studies, we assessed the TRIM26 KD efficiency by reverse transcription—quantitative polymerase chain reaction (qPCR) and western blotting. siRNA for TRIM26 (siTRIM26) significantly downregulated RNA and protein levels of TRIM26 in HepG2 cells expressing the HBV entry receptor, sodium taurocholate co-transporting polypeptide (NTCP) (HepG2-NTCP cells) (Figs. 1A,B and S6). HepG2-NTCP and PXB-cells, which are primary hepatocytes derived from humanized liver chimeric mice, were infected with HBV genotype C and transfected with siTRIM26, then evaluated the viral replication by measuring HBV DNA and HBs in the culture supernatants and 3.5 kb RNA and HBc in the cells. TRIM26 KD significantly attenuated HBV DNA, 3.5 kb RNA, HBs, and HBc in both cells (Figs. 1C,D, S1 and S12). To rule out the possibility that the effects of TRIM26 KD on HBV replication were off-target effects of siTRIM26, we employed another siRNA for TRIM26 (siTRIM26#2) to analyze its effect on HBV replication as well (Figs. S1 and S12). siTRIM26#2 as well as siTRIM26 reduced HBV DNA, 3.5 kb RNA, and HBc in HBV-infected HepG2-NTCP cells. Hence, the influences of TRIM26 KD on HBV replication were not off-target effects. For the sake of simplicity, we hereafter employed only siTRIM26 but not siTRIM26#2 for the following assays.

We also assessed how TRIM26 overexpression (OE) affects the HBV replication in HepG2-NTCP cells (Figs. S2 and S13). However, TRIM26 OE did not alter any of the parameters for HBV replication. This may indicate that additional TRIM26 does not have any effects although we cannot rule out the possibility that TRIM26 OE level was not enough to affect HBV replication.

### TRIM26 physically interacts with HBV core protein

Because TRIM26 had been reported to modulate either the host IFN response against virus infections or the functions of viral proteins, we investigated which molecular function was associated with the HBV replication^16–18^. Firstly, we examined whether TRIM26 downregulates IFN response associated with HBV infection (Fig. S3). In addition to type-I IFN, type-III IFN was also analyzed because both IFNs are similarly regulated by IRF3^20,21^. Each time period for the measurement was set within 48 h because IFN response against virus infection occurs at early time periods. Unexpectedly, IFNs and one of the interferon-stimulated genes (ISGs), CXCL10, were not increased by TRIM26 KD (Fig. S3). Hence, we considered that TRIM26-mediated promotion of HBV replication is independent of the downregulations of IFN responses.

**Figure 2:**
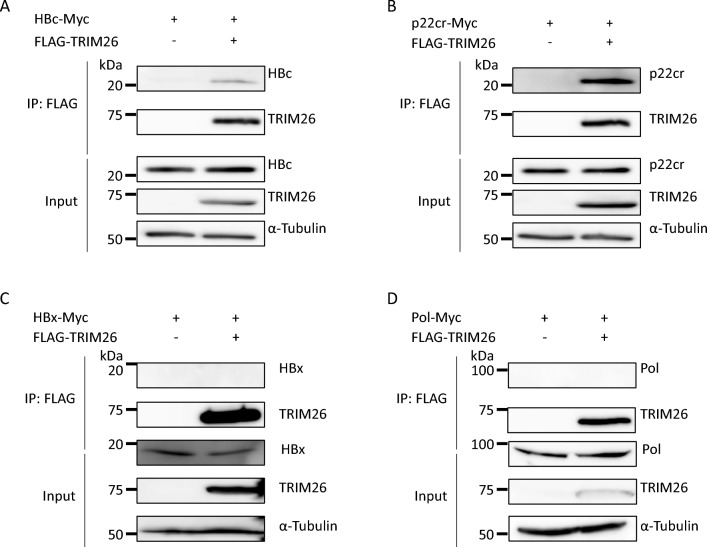
Overexpressed TRIM26 interacts with HBc in HEK293T cells. HEK293T cells were transiently transfected with the expression plasmids for (**A**) HBc-Myc, (**B**) p22cr-Myc, (**C**) HBx-Myc, or (**D**) Pol-Myc with or without FLAG-TRIM26. Cells were harvested at 48 hpt and the cell lysates were immunoprecipitated using an anti-FLAG antibody. Immunoprecipitated samples were analyzed by western blotting. The full-length images are shown in Fig. S7.

A previous study has reported that TRIM26 interacted with the HCV protein and promoted replication in an IFN-independent manner^18^. To examine the other possibility that TRIM26 modulates HBV protein function, we conducted co-immunoprecipitation (Co-IP) for TRIM26 and HBV proteins (Figs. 2 and S7). HEK293T cells were transfected with the expression plasmids for FLAG-tagged TRIM26 (FLAG-TRIM26) and Myc-tagged HBc (HBc-Myc), p22cr (the intracellular form of HBe) (p22cr-Myc), HBx (HBx-Myc), or polymerase (Pol) (Pol-Myc), then analyzed their interactions by Co-IP. Co-IP clearly demonstrated that TRIM26 interacted with HBc and p22cr, which share most amino acids except for the first 10 amino acids of p22cr, by binding to the common sites^22^. However, we excluded p22cr from further analysis because TRIM26 also affected precore-defective HBV (data not shown).

### Determination of the important domain for the interaction between TRIM26 and HBc

**Figure 3:**
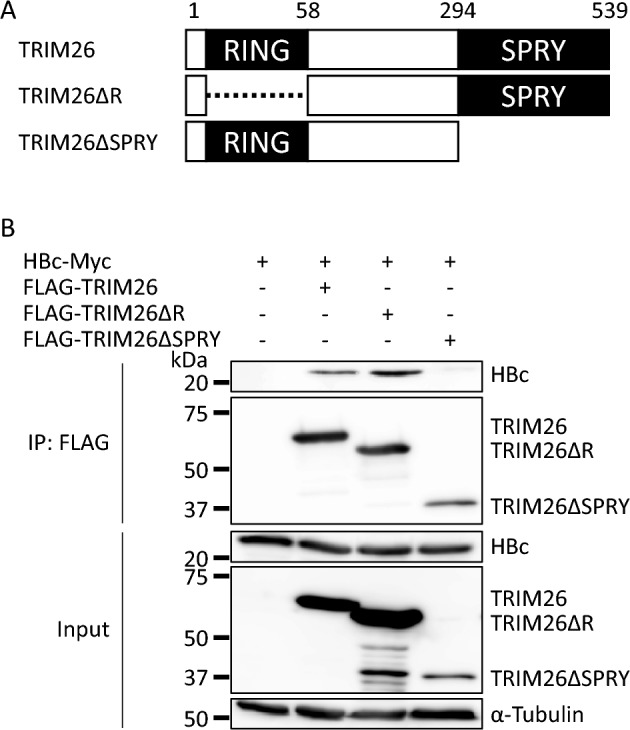
Determination of the important domain of TRIM26 for the interaction with HBc. (**A**) Schematic representation of deletion mutants of TRIM26 used in this study. (**B**) The expression plasmid for HBc-Myc was co-transfected with the expression plasmids for deletion mutants of FLAG-TRIM26. Cell lysates were subjected to immunoprecipitation and analyzed by western blotting. The full-length images are shown in Fig. S8.

To determine the domains that are important for this interaction, deletion mutants of TRIM26 were generated and analyzed by Co-IP in HEK293T cells (Figs. 3 and S8). Full-length TRIM26, RING domain-defective TRIM26 (TRIM26ΔR), and SPRY domain-defective TRIM26 (TRIM26ΔSPRY) were immunoprecipitated and their interactions with HBc were analyzed (Figs. 3 and S8). TRIM26 and TRIM26ΔR were equivalently precipitated with HBc, while TRIM26ΔSPRY showed the lower amount of precipitated HBc. This indicated that TRIM26 interacts with HBc through the SPRY domain.

### Endogenous TRIM26 interacts with HBc in hepatocytes

**Figure 4:**
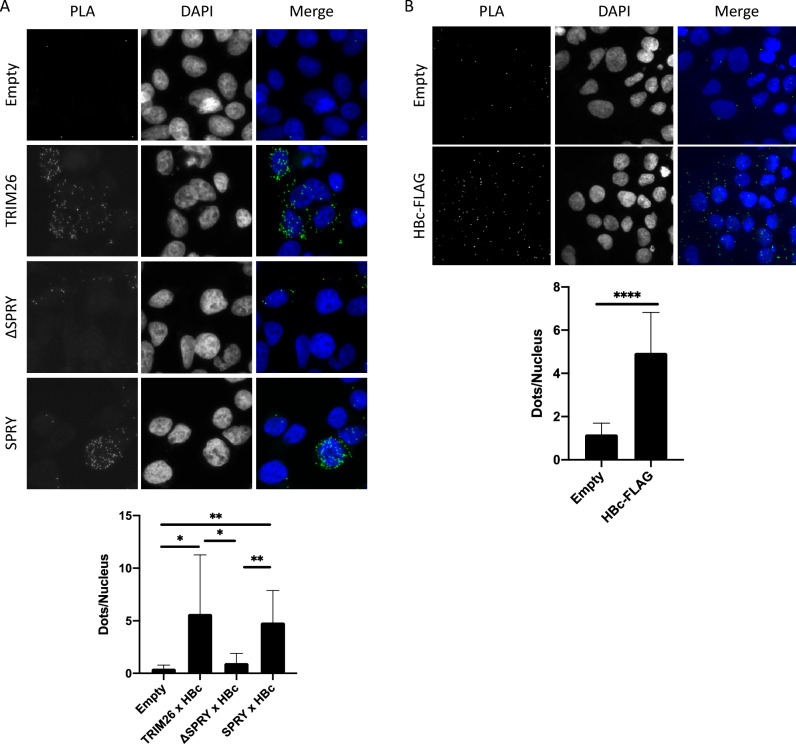
Proximity Ligation Assay (PLA) for the interaction between TRIM26 and HBc in hepatocytes. (**A**) The interaction between TRIM26 and HBc was analyzed by PLA in HEK293T cells transiently co-transfected with the expression plasmids for HBc-Myc and Empty, FLAG-TRIM26, FLAG-TRIM26ΔSPRY, or FLAG-SPRY. Antibodies against Myc-tag and FLAG-tag were used for PLA to examine the interaction between HBc and TRIM26 proteins. PLA dots of 91–126 cells (nucleus) from 10 different fields were counted using a confocal laser microscopy and the average numbers of PLA dots per nuclei were compared. (**B**) The interaction between HBc and endogenous TRIM26 was analyzed by PLA in Huh-7 and Huh-7/HBc-FLAG cells. Antibodies against FLAG-tag and TRIM26 were used for PLA. PLA dots of 643 Huh-7 cells and 444 Huh-7/HBc-FLAG cells from 10 different fields were counted and the average numbers were compared. Values are shown as the mean ± standard deviations of each field and all the significances were determined by student’s *t*-test and shown using asterisks (*P < 0.05, **P < 0.01, ****P < 0.0001).

We confirmed the intracellular interaction between TRIM26 and HBc by Proximity Ligation Assay (PLA) using transiently transfected FLAG-TRIM26 and HBc-Myc in HEK293T cells (Fig. 4A). The PLA signal was attenuated by the deletion of SPRY domain (ΔSPRY in Fig. 4A), whereas the solely expressed SPRY domain showed a PLA signal equivalent to that of full-length TRIM26 (SPRY in Fig. 4A). These results support the Co-IP results shown in Figs. 2 and 3 and indicate that the TRIM26 SPRY domain physically interacts with HBc. HBc also interacted with endogenous TRIM26 in Huh-7 cells stably transfected with FLAG-tagged HBc (HBc-FLAG) (Huh-7/HBc-FLAG cells) (Fig. 4B). These results confirmed that not only artificially expressed but also endogenous TRIM26 interacts with HBc in the hepatocytes.

### TRIM26 interrupts the ubiquitination and proteasome-dependent degradation of HBc

**Figure 5:**
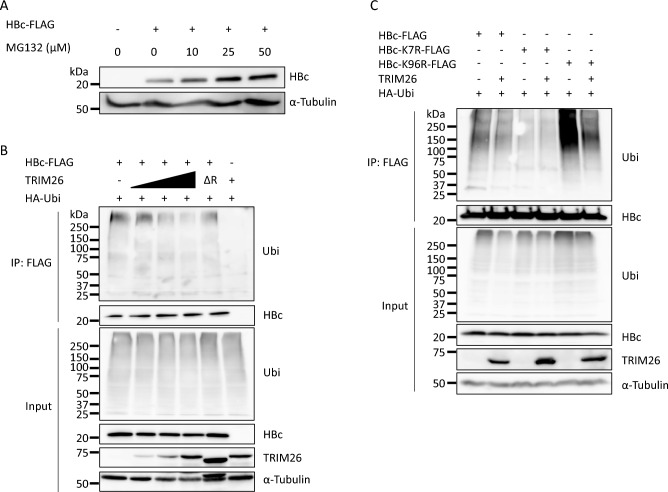
TRIM26 prevents the proteasome-dependent HBc degradation by inhibiting ubiquitination. (**A**) HEK293T cells were transiently transfected with the HBc-FLAG-expressing plasmid and treated with 25 μM MG132 for 4 h just before harvest. The cells were harvested at 48 hpt and HBc-FLAG protein levels were analyzed by western blotting. (**B**) HEK293T cells were co-transfected with the expression plasmids for HBc-FLAG, TRIM26, and HA-Ubi, treated with 25 μM MG132 for 4 h just before harvest, harvested at 48 hpt, and immunoprecipitated using an antibody against FLAG-tag. The ubiquitination status and proteins were analyzed by western blotting. The black triangle indicates the amount of TRIM26-expressing plasmid. ΔR: TRIM26ΔR mutant. (**C**) HEK293T cells were co-transfected with the expression plasmids for TRIM26, HA-Ubi, and HBc-FLAG or HBc-FLAG K to R mutants. The cells were treated with 25 μM MG132 for 4 h just before harvest, harvested at 48 hpt, and immunoprecipitated using an antibody against FLAG-tag. The proteins were analyzed by western blotting. We have reproduced each phenomenon at least 3 times and the figures show the representative data. The full-length images are shown in Fig. S9.

Since TRIM26 is an E3 ubiquitin ligase, we analyzed whether TRIM26 affects HBc ubiquitination. First, HEK293T cells transiently overexpressed HBc-FLAG were treated with various concentrations of MG132, a proteasome inhibitor, and subsequently analyzed the HBc-FLAG expression by western blotting (Figs. 5A and S9). The protein level of HBc-FLAG increased dose-dependently of MG132 treatment, indicating that HBc can be degraded in a proteasome-dependent manner as shown in the previous studies^23–25^. To examine the effect of TRIM26 on HBc ubiquitination, an in vivo ubiquitination assay was conducted by transfecting HEK293T cells with the expression plasmids for HBc-FLAG, HA-tagged ubiquitin (HA-Ubi), and TRIM26 without any tag (Figs. 5B and S9). Unexpectedly, HBc was intensely ubiquitinated in the absence of TRIM26 OE, whereas ubiquitination was impaired by the increase of TRIM26. Interestingly, TRIM26ΔR has little effect on HBc ubiquitination (Figs. 5B and S9), suggesting that TRIM26 inhibits HBc ubiquitination through the RING domain. HBc contains two lysine residues, K7 and K96, which can be the targets of ubiquitination^26^. We next assessed which residue is associated with the inhibitory function of TRIM26 (Figs. 5C and S9). By comparing the ubiquitination of WT, K7R, and K96R, K7 should be the main ubiquitination residue as reported previously because K7R lost the ubiquitination^26^. Besides, the ubiquitination of K96R as well as WT was reduced by TRIM26 transfection. These results indicate that TRIM26 inhibits the ubiquitination of K7.

**Figure 6:**
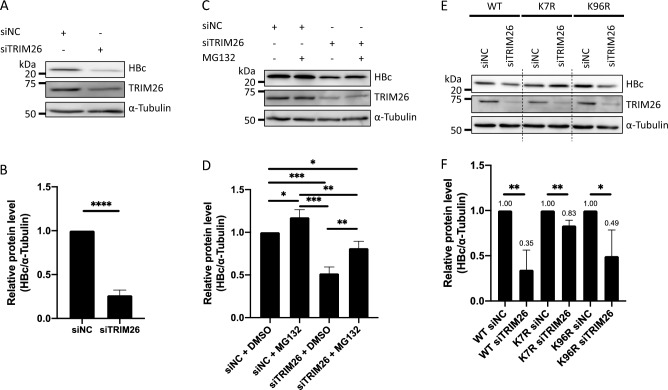
TRIM26 prevents HBc from the proteasome-dependent degradation. (**A**) Huh-7/HBc-FLAG cells were transfected with siNC or siTRIM26 and the proteins were analyzed by western blotting at 96 hpt. (**B**) The protein levels of HBc-FLAG were normalized by the protein levels of α-Tubulin and shown as the mean ± standard deviations of three independent experiments. (**C**) Huh-7/HBc-FLAG cells were transfected with siNC or siTRIM26 and treated with or without 25 μM MG132 for 4 h just before harvest. The cells were harvested at 96 hpt of siRNA to analyze the proteins by western blotting. (**D**) The protein levels of HBc-FLAG were normalized by the protein levels of α-Tubulin and shown as the mean ± standard deviations of three independent experiments. (**E**) Huh-7 stably transfected with the expression plasmids for HBc-FLAG or its K to R mutants were transfected with siNC or siTRIM26. The cells were harvested at 96 hpt and the proteins were analyzed by western blot. (**F**) The protein levels of HBc-FLAG were normalized by the protein levels of α-Tubulin and shown as the mean ± standard deviations of three independent experiments. All the significances were determined by the student's *t*-test and shown using the asterisks (*P < 0.05, **P < 0.01, ***P < 0.001, ****P < 0.0001). The full-length images are shown in Fig. S10.

Next, we investigated how TRIM26 affects the protein levels of HBc. Huh-7/Core-FLAG cells were subjected to siRNA-mediated TRIM26 KD and the protein levels of HBc were measured by western blotting. HBc was significantly reduced by TRIM26 KD (Fig. 6A,B and S10) and restored by MG132 treatment (Figs. 6C,D and S10). Consistent with the ubiquitination levels, the K7R mutant was less affected by TRIM26 KD than the WT and K96R mutants (Figs. 6E,F and S10), indicating that TRIM26 prevents the proteasome-dependent HBc degradation. We also examined whether TRIM26 OE altered the HBc levels in Huh-7/HBc-FLAG cells by transient transfection of the TRIM26 expression plasmid. HBc was significantly upregulated by TRIM26 OE, although the difference was not drastic (Figs. S4 and S14). The TRIM26-mediated upregulation of HBc was impaired by deletions of the important domains of TRIM26 in Huh-7/HBc-FLAG cells (Figs. S5 and S15). These results confirmed that TRIM26 modestly upregulates HBc and all the domains of TRIM26 are required for the phenomenon; however, the influence of TRIM26 OE on HBc level was limited.

**Figure 7:**
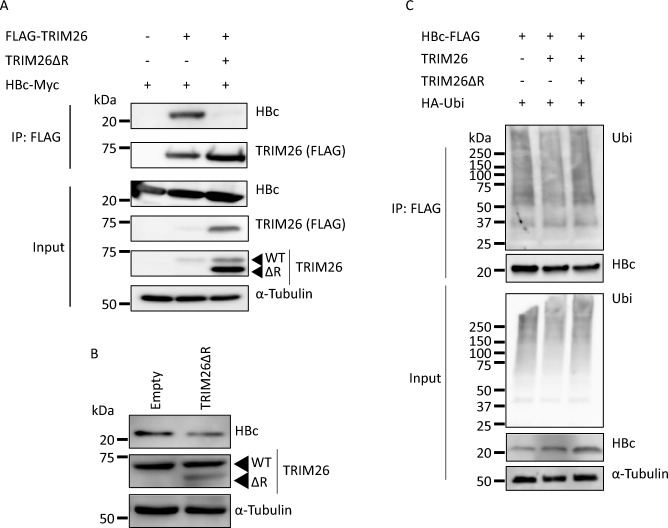
TRIM26ΔR interferes with the function of TRIM26. (**A**) HEK293T cells were co-transfected with the expression plasmids for FLAG-TRIM26, TRIM26ΔR, and HBc-Myc. The cells were harvested at 48 hpt and immunoprecipitated using anti-FLAG antibody. Indicated proteins were detected by western blotting. (**B**) Huh-7/HBc-FLAG cells were transduced with a lentiviral vector for TRIM26ΔR, and the protein level of HBc-FLAG was examined by western blotting. Empty indicates the control cells transduced with a lentiviral vector containing no insert. The photo for HBc was obtained from the different blot of TRIM26 and α-Tubulin. (**C**) HEK293T cells were co-transfected with the expression plasmids for TRIM26, TRIM26ΔR, HA-Ubi, and HBc-FLAG as indicated in the figure. The ubiquitination levels of HBc-FLAG were analyzed as described elsewhere. We have reproduced each phenomenon at least 3 times, and the figures show the representative data. The full-length images are shown in Fig. S11.

Although TRIM26 physically interacts with HBc, it remains unclear whether physical interactions are required to prevent the proteasome-dependent degradation of HBc. TRIM26ΔR lost the ability to interrupt HBc ubiquitination, but it still interacts with HBc (Figs. 3B, 5B, S8 and S9). Therefore, we presumed that TRIM26ΔR might act as a dominant-negative form of TRIM26 in the interaction between TRIM26 and HBc. A competitive Co-IP study revealed that TRIM26ΔR sequestered TRIM26 from HBc in HEK293T cells (Figs. 7A and S11). We also analyzed the influence of TRIM26ΔR on HBc level in Huh-7/HBc-FLAG cells transduced with or without TRIM26ΔR and revealed that HBc level decreased in the TRIM26ΔR-expressing cells (Figs. 7B and S11). Furthermore, TRIM26-mediated impairment of HBc ubiquitination was restored by competitively expressed TRIM26ΔR (Figs. 7C and S11). These results demonstrated that TRIM26ΔR interferes the interaction between TRIM26 and HBc, thereby promoting HBc ubiquitination and degradation.

## Discussion

**Figure 8:**
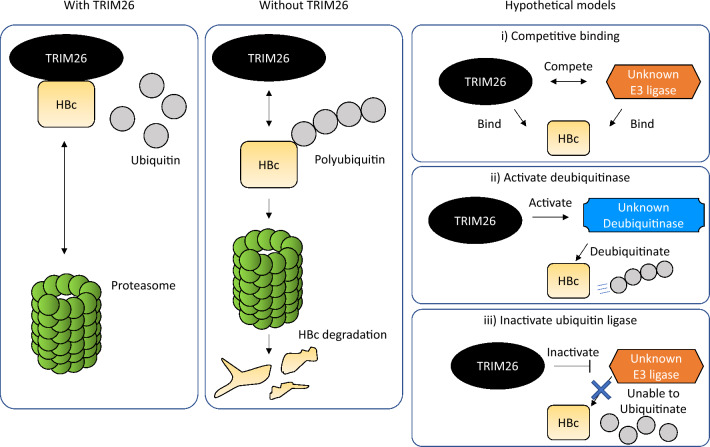
Schematics for the hypothetical mechanism of TRIM26-mediated HBc deubiquitination. HBc is prevented from ubiquitination and proteasome-dependent degradation in the presence of TRIM26, while HBc is subjected to polyubiquitination and proteasome-dependent degradation in the absence of TRIM26. Three hypothetical mechanistic models can be considered for the TRIM26-mediated inhibition of the proteasome-dependent HBc degradation. i) TRIM26 sequestrates an unknown E3 ubiquitin ligase from HBc by competitive binding. ii) TRIM26 ubiquitinates an unknown HBc-specific deubiquitinase to activate the catalysis. iii) TRIM26 inactivates the catalysis of an unknown HBc-specific E3 ubiquitin ligase.

In this study, we showed that the interaction between TRIM26 and HBc is important for preventing the proteasome-dependent HBc degradation. This inhibitory effect was also attributed to the RING domain of TRIM26, which is responsible for its E3 ubiquitin ligase activity^18^. Surprisingly, TRIM26 does not ubiquitinate HBc itself, but prevents HBc ubiquitination. Several studies have shown that TRIM proteins inactivate other E3 ubiquitin ligases to maintain the ubiquitination balance of substrates in various ways^27–29^. TRIM26 interrupts WWP2, a ubiquitin ligase, to prevent SOX2 protein from ubiquitination and degradation in glioblastoma stem cells (GSCs), which maintains the undifferentiated status of GSCs^27^. Although the details are not well understood, this phenomenon is based on the competitive binding of TRIM26 and WWP2 to the SOX2 protein. TRIM27 interacts with and ubiquitinates ubiquitin-specific protease (USP) 7 to activate its deubiquitination activity. Activated USP7 deubiquitinates receptor-interacting protein 1, which promotes tumor necrosis factor-alpha^28^. TRIM41 ubiquitinates ZSCAN21, an important transcription factor for the α-synuclein gene (SNCA), to reduce the risk for Parkinson disease (PD)^29^. However, TRIM17 disturbs TRIM41 through an unknown mechanism, which may stabilize ZSCAN21, increase SNCA transcription, and progress to PD. Thus, at least three different molecular interactions can be considered as the following mechanism for TRIM26-mediated inhibition of HBc ubiquitination: i) TRIM26 sequesters an unknown E3 ubiquitin ligase from HBc by competitive binding, ii) TRIM26 ubiquitinates an unknown HBc-specific deubiquitinase to activate catalysis, and iii) TRIM26 inactivates the catalysis of an unknown HBc-specific E3 ubiquitin ligase (Fig. 8). These precise mechanisms should be examined in the future.

Our observations are consistent with the previous studies that HBc is degraded in a proteasome-dependent manner, which were confirmed by treatment of HBc-expressing cells with MG132 and/or Lactacystin^23–25^. Besides, Langerová et al.^26^ reported that K7 but not K96 was predominantly ubiquitinated as we confirmed in this study. Garcia et al.^30^ reported that K96 is not involved in the ubiquitin-associated HBV replication cycle. These studies may support our findings that K7 ubiquitination is associated with the proteasome-dependent degradation of HBc, which is inhibited by TRIM26.

The SPRY domains of TRIM proteins are important for protein–protein interactions^31^. TRIM26 interacts with multiple proteins such as TBK1, DEAD-Box Helicase 41, sex-determining region Y-box2, and HCV NS5B through the SPRY domain of TRIM26^18,27,32,33^. These studies support our finding that the TRIM26 SPRY domain is required for the interaction between TRIM26 and HBc.

TRIM26 was suggested as the host factor that promotes viral replication by degrading nuclear IRF3 to inhibit the type I IFN response upon viral infection^16,17^. In these studies, TRIM26 was induced by IFN against viruses, including Sendai virus, VSV, and HSV-2 in the immune cells. Then, TRIM26 is translocated into the nucleus to ubiquitinate and degrade phosphorylated IRF3 in a proteasome-dependent manner, which downregulates the IFN response and enhances viral replication. On the other hand, based on our findings, TRIM26 was not induced by HBV infection in the hepatocytes (data not shown), and besides, TRIM26 OE had limited impact on HBV replication and HBc stability. This indicates that the steady state of TRIM26 circumstantially supports HBV replication. Furthermore, TRIM26 was not involved in the IFN response against HBV infection in HepG2-NTCP cells (Fig. S3). This may be because HBV is a stealth virus that does not induce IFNs and ISGs in hepatocytes^34^.

Recently, Luo et al. have reported that TRIM26 inhibits HBV replication by promoting HBx degradation, which may be affected by IFN treatment^35^. They found that TRIM26 OE impaired, whereas TRIM26 KD enhanced HBV replication. They also suggested that TRIM26 physically interacts with HBx to ubiquitinate and degrade it in a proteasome-dependent manner. Their finding regarding the role of TRIM26 in HBV replication seems to be opposite to ours. We are not sure of the reason yet, however, further studies should be conducted to resolve these controversial results.

As shown in this study, the short-term suppression of TRIM26 by siRNA did not show robust effect on HBV replication; however, a long-term inhibition of the interaction between TRIM26 and HBc could suppress HBV replication more efficiently. These issues should be addressed in future studies.

In summary, we identified a novel mechanism of HBV replication; HBV utilizes TRIM26 to evade the proteasome-dependent degradation of HBc. Our findings may be helpful for developing new therapeutic strategies for chronic HBV infection in the future.

## Methods

### Cell culture

Human hepatoma cell lines HepG2 and Huh-7 cells were obtained from American Type Culture Collection (Manassas, VA, USA) and the Health Science Research Resources Bank (Osaka, Japan), respectively. HepG2 and Huh-7 cells were maintained in Dulbecco’s modified Eagle’s medium (DMEM) with a low glucose concentration (1 g/L) (FUJIFILM Wako Pure Chemical Corporation, Osaka, Japan) supplemented by 10% fetal calf serum (FCS) at 37 °C in a humidified atmosphere containing 5% CO_2_. Human embryonic kidney (HEK) 293T cells were maintained in DMEM with a high glucose concentration (4.5 g/L) (FUJIFILM Wako Pure Chemical Corporation) supplemented by 10% FCS at 37 °C in a humidified atmosphere containing 5% CO_2_. HepG2-NTCP cells were generated by inoculating HepG2 cells with a lentiviral vector which contains NTCP and neomycin-resistance genes. The transduced cells were selected by 600 μg/ml G418 (Tokyo chemical industry, Tokyo, Japan) and prepared for HBV inoculation experiments. Human primary hepatocytes, PXB-cells (PhoenixBio, Hiroshima, Japan), were maintained in the culture medium for PXB-cells (PhoenixBio) according to the manufacturer’s instructions. Huh-7/HBc-FLAG cells were transduced with a lentiviral vector (pLVSIN-EF1α-Hyg, TaKaRa) containing TRIM26ΔR and selected by 250 μg/ml hygromycin B to establish the cells stably express TRIM26ΔR (Fig. 7B).

### Virus preparation

HBV stocks were prepared by transfection of a plasmid containing 1.3-fold HBV genotype C genome as described previously^36^. Virus titer was determined by qPCR with a primer pair for HBV DNA (Table S1) and calculated as copy numbers in 1 ml virus stock solution. The details for the procedures of qPCR are described in the subsection for qPCR.

### HBV inoculation

HBV inoculation was carried out as described elsewhere^19^. In brief, HBV stock solution adjusted to desired multiplicity of infection was mixed with 2% dimethyl sulfoxide (DMSO) and 4% polyethylene glycol 8000 and incubated with the cells stably express NTCP gene at 37 °C for approximately 24 h. The inoculum was removed, and the cells were washed five times with phosphate buffered saline (-) followed by incubation in a growth medium containing 2% DMSO prior to being analyzed. The growth medium was replaced with the fresh one every two or three days.

### Isolation of HBV DNA

HBV DNA was isolated from the culture supernatant using SMITEST EX-R&D (Medical & Biological Laboratories, Tokyo, Japan) or ISOSPIN Blood & Plasma DNA (NIPPON GENE CO., LTD, Tokyo, Japan).

### Synthesis of complementary (c) DNA

Total RNA was isolated from culture cells using ISOGEN II or ISOSPIN Cell & Tissue RNA (NIPPON GENE CO., LTD). cDNA was synthesized from 0.5 to 1 μg total RNA using SuperScript IV (Thermo Fisher Scientific, Waltham, MA, USA) with Random Primer (TaKaRa) according to the manufacturer’s instructions.

### Co-IP and western blotting

HEK293T cells were transfected with various combinations of plasmids as indicated in each figure by a calcium-phosphate transfection method. Shortly, a total of around 30 μg plasmids were mixed with 125 mM CaCl_2_ and 1 × BES-buffered saline pH6.95, incubated at room temperature for 15 min, then added to the cells in a dropwise manner. Medium was replaced with the fresh growth medium at 24 hpt and additionally incubated for 24 more hours. At 48 hpt, cells were harvested with a NP-40 buffer (50 mM Hepes pH7.5, 150 mM NaCl, 0.5% NP-40) supplemented with a protease inhibitor cocktail (nacalai tesque, Kyoto, Japan). The lysates were precleared using SureBeads Protein G Magnetic Beads (Bio-Rad, Hercules, CA, USA) and precleared lysates containing 1–2 mg protein was subjected to IP with the antibody-conjugated magnetic beads as indicated by rotating at room temperature for 1 h. The beads were washed 5 times with the NP-40 buffer and the immunoprecipitated samples were eluted in 2 × Laemmli’s sample buffer by boiling at 100 °C for 5 min. The eluted samples were analyzed by western blotting as follows. Samples were electrophoresed in SDS-PAGE and transferred onto PVDF membrane (Immobilon-P, 0.45 μm pore-sized, Merck, Darmstadt, Germany), blocked with Tris-buffered saline (TBS) pH 7.5 containing 10w/v% of skim milk, reacted with a primary antibody and a secondary antibody followed by development of the signal using ImmunoStar Zeta (FUJIFILM Wako Pure Chemical Corporation). The signal was analyzed by a chemiluminescence detection system ImageQuant LAS 500 (Cytiva, Tokyo, Japan). The antibodies used in this study are listed in Table S2. The in-house anti-HBc mAb used in Figs. S1, S2, S12 and S13 was generated in a hybridoma cell line established from a mouse immunized with HBc antigen derived from Dane particle.

### MG132 treatment

Cells were treated with a proteasome inhibitor, MG132 (Chemscene LLC, Monmouth Junction, NJ, USA), at the indicated concentration during the indicated time just before harvest. Samples were subjected to in vivo ubiquitination assay or western blotting.

### In vivo ubiquitination assay

HEK293T cells were transiently transfected with the expression plasmids for FLAG-tagged Core, TRIM26, or their mutants together with HA-tagged ubiquitin using Lipofectamine3000 transfection reagent. At 44 hpt, cells were treated with 25 μM MG132 for 4 h, subsequently harvested with a ubiquitin lysis buffer (2% SDS, 150 mM NaCl, 10 mM Tris–HCl, pH 8.0) supplemented with a protease inhibitors cocktail and 100 mM N-ethylmaleimide (NEM) at 48 hpt, then denatured by boiling at 100 °C for 10 min. The lysates were tenfold diluted with a ubiquitin dilution buffer (10 mM Tris–HCl, pH 8.0, 150 mM NaCl, 2 mM EDTA, 1% Triton-X100) supplemented with a protease inhibitors cocktail and 100 mM NEM, rotated for 30 min at 4 °C, then centrifuged to collect the supernatants. Between 1 to 1.5 mg of the supernatants were subjected to immunoprecipitation using an anti-FLAG-tag antibody at 4 °C overnight. Next morning, SureBeads Protein G Magnetic Beads were added and rotated at 4 °C for 1 h, then washed five times with the ubiquitin dilution buffer and boiled at 70 °C for 10 min in a 1 × Laemmli’s sample buffer to elute the immunoprecipitated samples. Samples were analyzed by the western blotting as described above in the subsection of Co-IP and western blotting.

### qPCR

Isolated HBV DNAs and synthesized cDNAs were analyzed by qPCR as described previously with modifications^37^. Briefly, HBV DNA or cDNA were mixed with primer pairs listed in Table S1 and GeneAce SYBR qPCR Mix α (NIPPON GENE CO., LTD) with, then subsequently analyzed using an Applied Biosystems 7900HT Fast Real Time PCR System (Thermo Fisher Scientific). The thermal cycling condition was 95 °C for 10 min and 45 cycles of 95 °C for 30 s and 60 °C for 1 min. Standard curves were calculated using the Ct values of the tenfold serially diluted standard plasmids which contain the sequences of the amplicons.

### Plasmid construction

An expression plasmid for TRIM26 was purchased from Origene (Rockville, MD, USA) and TRIM26 coding sequence was transferred into pCMV-FLAG-Myc-22 (Merck) with a stop codon before Myc-tag sequence by normal molecular biology methods. The deletion mutants of TRIM26 fragments were amplified using PrimeStar Max DNA Polymerase (TaKaRa, Shiga, Japan) and cloned into pCMV-FLAG-Myc-22 using In-Fusion Snap Assembly Master Mix (TaKaRa)^38^. HBc, HBe, HBx, and HBV Pol were amplified from the extracted DNA of HepG2.2.15 using PrimeStar Max DNA Polymerase and cloned into pCMV-FLAG-Myc-22 to add a c-Myc-tag at the C-termini. The Myc-tagged HBV protein sequences were transferred into phCMV-1 (Genlantis, San Diego, CA, USA). HBc deletion mutants cloned in phCMV-1 were generated using In-Fusion Snap Assembly Master Mix. All the plasmids used in this study were approved by the committee for genetic recombination experiments at Jichi Medical University.

### siRNA-mediated TRIM26 KD

TRIM26 KD was carried out by transfection of siRNA for TRIM26 (siTRIM26: Santa Cruz Biotechnology, TX, USA, or siTRIM26#2: 5’- CCGGAGAAUUCUCAGAUAA -3′^39^). Negative control #1 siRNA (Thermo Fisher Scientific) was used as the negative control siRNA (siNC). The transfection was conducted using Lipofectamine RNAiMAX transfection reagent (Themo Fisher Scientific) in accordance with the manufacturer’s instructions with slight modifications. Briefly, 16 pmol siRNA was mixed with 4.8 μl LipofectamineRNAiMAX in 40 μl OPTI-MEM (Thermo Fisher Scientific) and incubated at RT for 15 min, then subsequently dispensed onto HepG2-NTCP or Huh-7 cells cultured in 24-well plates. The cells and culture supernatants were harvested 48–96 hpt depending on the purpose of the experiment.

### PLA

PLA was conducted as previously described with some modifications^40^. Cells were fixed with 4% paraformaldehyde at room temperature for 15 min, then subsequently permeabilized with 0.5% Triton-X100 in phosphate buffered saline (PBS) (-) for 5 min. After washing the cells with PBS (-) three times, cells were blocked with the blocking solution included in the PLA kit (Duolink In Situ Probe, Merck) at 37 °C for 1 h, followed by the reaction with the primary antibodies at 4 °C, overnight. The used antibodies are listed in Table S2. The following procedures were carried out according to the manufacturer’s instructions. Images were acquired from randomly chosen multiple fields using a confocal laser scanning biological microscope (FV1000, Olympus, Tokyo, Japan) and analyzed with ImageJ. Values are calculated and shown as PLA dots per nucleus according to the manufacturer’s instructions.

### Enzyme-linked immunosorbent assay (ELISA) for HBsAg

HBsAg was measured using Hepatitis B surface antigen ELISA kit (Abnova (Taiwan) Corporation, Taoyuan City, Taiwan) according to the manufacturer’s instructions.

### Statistics

All the significances were considered as *P* < 0.05 and determined by a two-tailed paired or unpaired student’s *t*-test using GraphPad Prism 8 (GraphPad Software, San Diego, CA, USA).

### Supplementary Information

Supplementary Information.

## Data Availability

All data generated or analyzed during this study are included in this published article and its Supplementary Information files.
