# Supplementary material for: TRIM26 positively affects hepatitis B virus replication by inhibiting proteasome-dependent degradation of viral core protein
Source: Sci Rep. 2023 Aug 21;13:13584. doi: 10.1038/s41598-023-40688-3 (PMC10442393; doi:10.1038/s41598-023-40688-3)
Supplement: Supplementary file 1 — Supplementary Information. [file 41598_2023_40688_MOESM1_ESM.docx]

**Supplementary document**

**TRIM26 positively affects hepatitis B virus replication by inhibiting proteasome-dependent degradation of viral core protein**

**Yuki Nakaya**^1, *^, **Tsutomu Nishizawa**^1^, **Hironori Nishitsuji**^2^, **Hiromi Morita**^1^, **Tomoko Yamagata**^1^, **Daichi Onomura**^1^, **Kazumoto Murata**^1, +^

^1^Division of Virology, Department of Infection and Immunity, Jichi Medical University, Shimotsuke, 329-0498, Japan, ^2^ Department of Virology and Parasitology, School of Medicine, Fujita Health University, Toyoake, 470-1192, Japan

^*^nakaya.yuki@jichi.ac.jp

^+^kmurata@jichi.ac.jp

**Supplementary Tables**

**Table S1. Primer pairs used for qPCR in this study**

| Target gene | Strand | Sequence |
| --- | --- | --- |
| IFN-β | F | 5’- CTTGGATTCCTACAAAGAAGCAGC -3’ |
|  | R | 5’- TCCTCCTTCTGGAACTGCTGCA -3’ |
| IFN-λ1 | F | 5’- AACTGGGAAGGGCTGCCACATT -3’ |
|  | R | 5’- GGAAGACAGGAGAGCTGCAACT-3’ |
| CXCL10 | F | 5’- GTGGCATTCAAGGAGTACCTC -3’ |
|  | R | 5’- TGATGGCCTTCGATTCTGGATT -3’ |
| TRIM26 | F | 5’- GAACCACCTGAGTACCCTAAGG -3’ |
|  | R | 5’- CTCAGCCACAATGTACTGCCTC -3’ |
| HBV DNA | F | 5’- AGACTCGTGGTGGACTTCTCTCA -3’ |
|  | R | 5’- TGAGGCATAGCAGCAGGATG -3’ |
| 3.5 kb RNA | F | 5’- GACCACCAAATGCCCCTATC -3’ |
|  | R | 5’- GATTGAGATCTTCTGCGACGC -3’ |
| GAPDH | F | 5’- GTCTCCTCTGACTTCAACAGCG -3’ |
|  | R | 5’- ACCACCCTGTTGCTGTAGCCAA -3’ |
|  |  |  |

**Table S2. Antibodies used in this study**

| Antibody name | Target | Vendor | Catalog no |
| --- | --- | --- | --- |
| TRIM26 (A-7) | TRIM26 | Sant Cruz Biotechnology, Inc. | sc-393832 |
| TRIM26 Polyclonal antibody | TRIM26 | Proteintech | 27013-1-AP |
| Anti-HBc mAb | HBc | Prepared in our lab |  |
| Anti-Myc-tag mAb | Myc-tag | MBL | N192-3 |
| Myc-Tag (71D10) Rabbit mAb | Myc-tag | Cell Signaling Technology | 2278 |
| Monoclonal ANTI-FLAG M2 | FLAG-tag | Merck | F1804 |
| DYKDDDDK tag Polyclonal antibody | FLAG-tag | Proteintech | 20543-1-AP |
| HA tag Rabbit Polyclonal antibody | HA-tag | Proteintech | 51064-2-AP |
| Anti α-Tubulin, mAb | α-Tubulin | FUJIFILM Wako Pure Chemical | 013-25033 |

**Supplementary Figures**


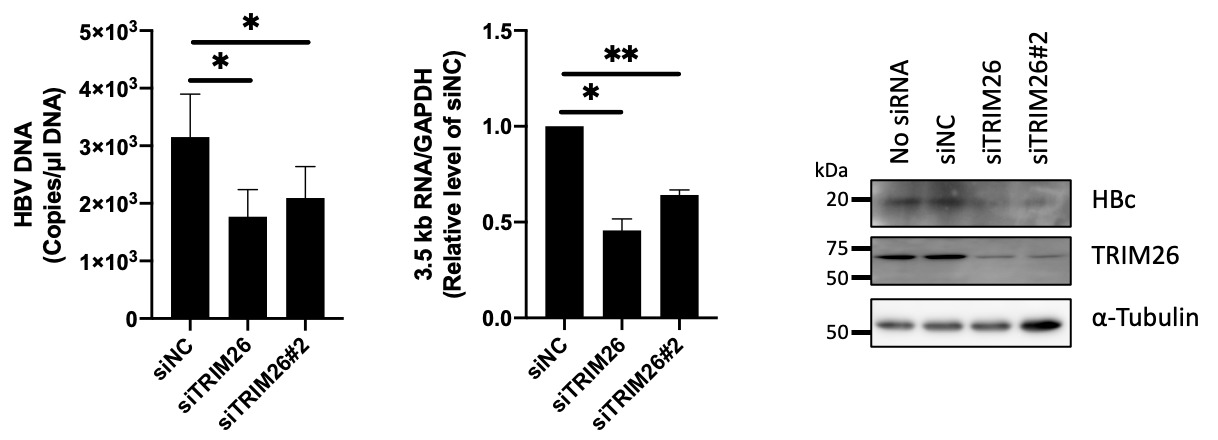


**Figure S1. Analysis of the effects of TRIM26 KD with another siRNA on HBV replication in HepG2-NTCP cells.** HepG2-NTCP cells inoculated with HBV at the MOI of 50 GEq/cell were transfected with siNC, siTRIM26, or TRIM26#2. Cells were harvested at 96 h post transfection to analyze the HBV DNA, 3.5 kb RNA, and HBc levels. 3.5 kb RNA level was normalized by that of GAPDH. Values are shown as the mean ± standard deviations of three independent experiments. The pictures of the western blotting are the representative data. Significances were determined by student’s *t*-test using GraphPad Prism 8 and indicated with asterisks (**P* < 0.05, ***P* < 0.01). The full-length images are shown in Fig. S12.


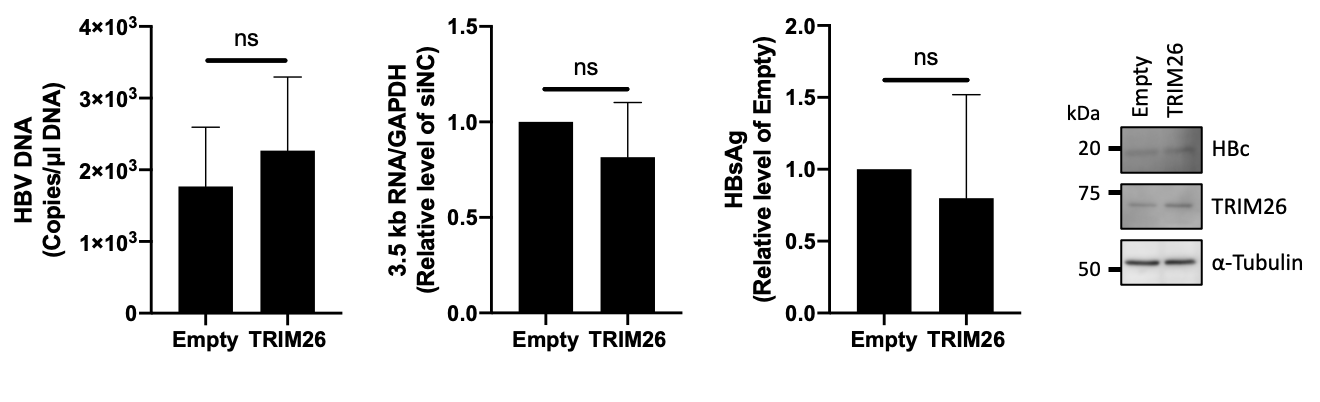


**Figure S2. Analysis of the effect of TRIM26 OE on HBV replication in HepG2-NTCP cells.** HepG2-NTCP cells inoculated with HBV at the MOI of 50 GEq/cell were transfected with Empty or TRIM26 expression plasmids. Cells were harvested at 72 h post transfection to analyze the HBV DNA, 3.5 kb RNA, HBs, and HBc levels. 3.5 kb RNA level was normalized by that of GAPDH. Values are shown as the mean ± standard deviations of three independent experiments. Significances were determined by student’s *t*-test using GraphPad Prism 8 but no significance was observed. The full-length images are shown in Fig. S13.

**
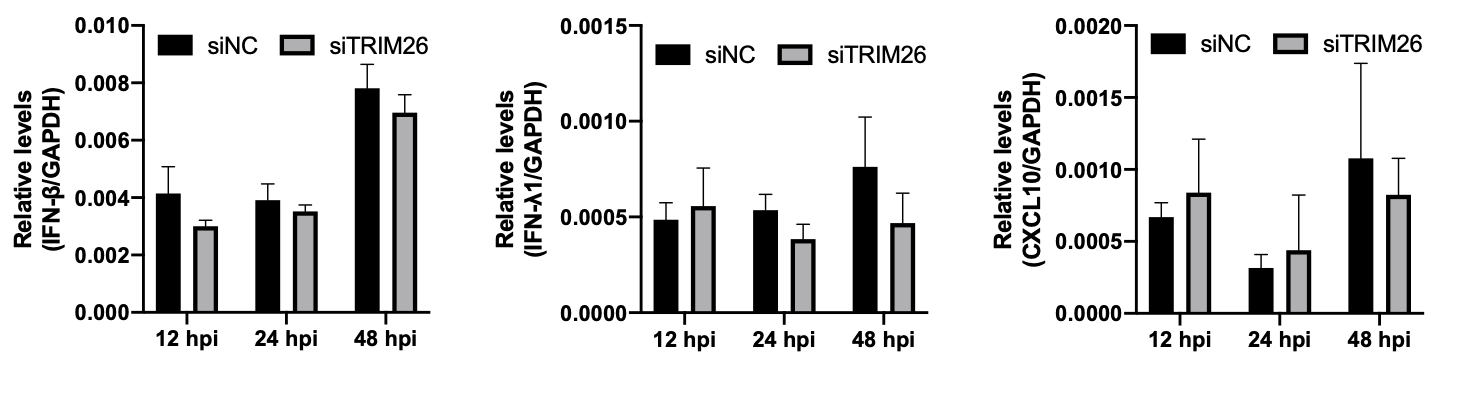
**

**Figure S3. Analysis of the effect of TRIM26 KD on IFN response against HBV infection in HepG2-NTCP cells.** HepG2-NTCP cells transfected with siNC or siTRIM26 were inoculated with HBV at the MOI of 500 GEq/cell. Cells were harvested at 12, 24, and 48 hpi to analyze the expression levels of the indicated genes. Gene levels were normalized by GAPDH and shown as the mean ± standard deviations of three independently infected samples. No significance was observed by student’s *t*-test performed by GraphPad Prism 8.
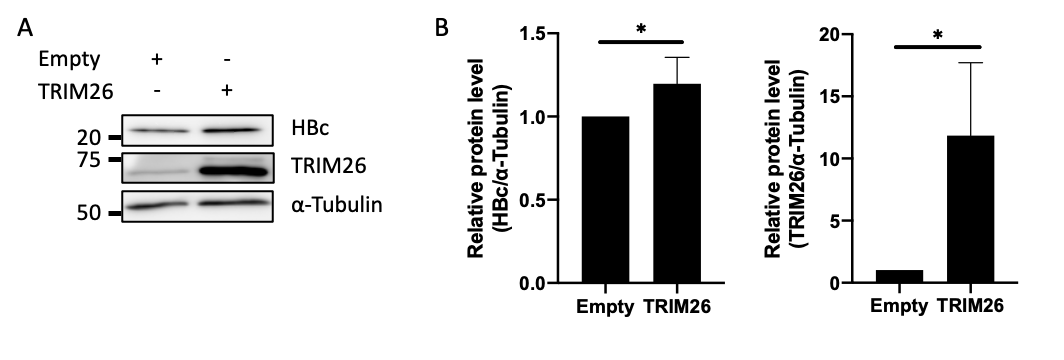


**Figure S4. TRIM26 overexpression in Huh-7 cells tends to increase HBV core protein.** Huh-7 cells stably express FLAG-tagged HBV core protein were transfected with the TRIM26 expression plasmid or its backbone empty plasmid using Lipofectamine3000 according to the manufacturer’s instructions. Cells were harvested with a NP-40 buffer (50 mM Hepes pH7.5, 150 mM NaCl, 0.5% NP-40) at 48 hpt and analyzed by western blotting. (A) Core protein, TRIM26, and α-Tubulin were detected by the monoclonal antibodies for FLAG-tag, TRIM26, and α-Tubulin, respectively (Table S2). The full-length blots are shown in Fig. S14 (B) Each protein level was analyzed by Image J, normalized by the level of α-Tubulin, and shown as the ratio to empty vector-transfected cells. Values are shown as the mean ± standard deviations of four independent experiments. Significances were determined by student’s *t*-test using GraphPad Prism 8 and exhibited with an asterisk (**P* < 0.05).


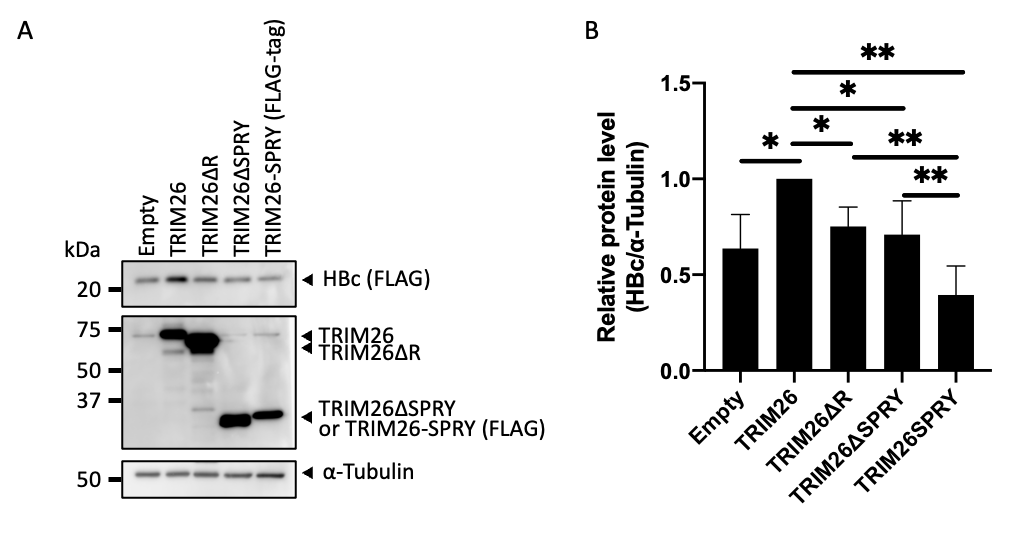


**Figure S5. TRIM26-mediated HBc upregulation is attenuated by the deletions of important domains of TRIM26.** Huh-7 cells stably express FLAG-tagged HBV core protein were transfected with the Empty, TRIM26, TRIM26ΔR, TRIM26ΔSPRY, or TRIM26-SPRY (FLAG-tagged) expression plasmids using Lipofectamine3000 according to the manufacturer’s instructions. Cells were harvested with a NP-40 buffer (50 mM Hepes pH7.5, 150 mM NaCl, 0.5% NP-40) at 48 hpt and analyzed by western blotting. (A) Core protein, TRIM26, and α-Tubulin were detected by the monoclonal antibodies for FLAG-tag, TRIM26, and α-Tubulin, respectively (Table S2). The full-length images are shown in Fig. S15. (B) Each protein level was analyzed by Image J, normalized by the level of α-Tubulin, and shown as the ratio to the cells transfected with TRIM26. Values are shown as the mean ± standard deviations of three independent experiments. Significances were determined by student’s *t*-test using GraphPad Prism 8 and exhibited with asterisks (**P* < 0.05, ***P* < 0.01).

**
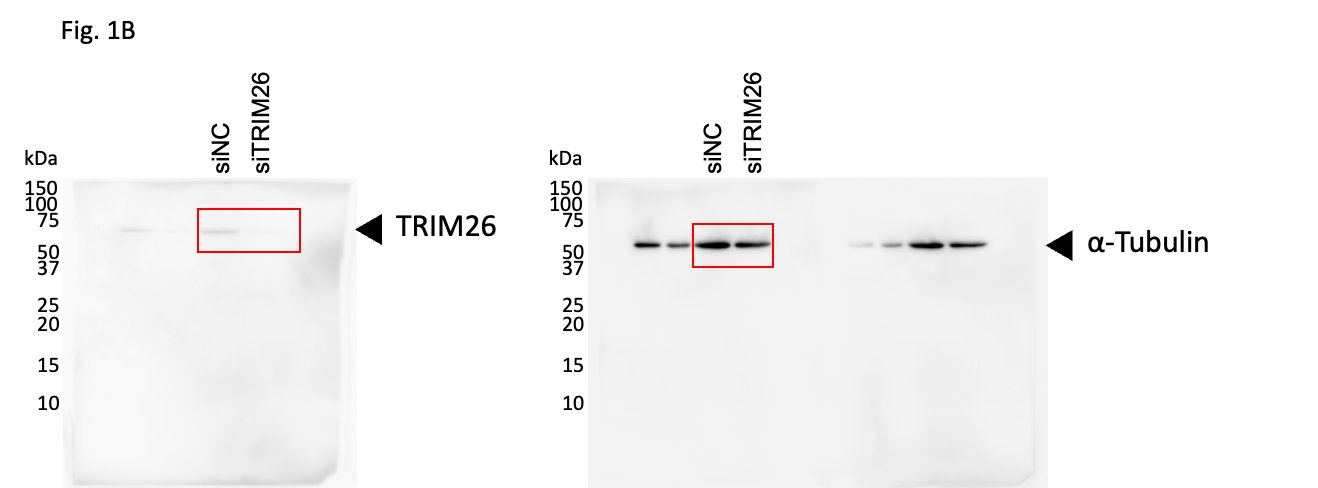
**

**Figure S6. Full-length blots for images of Figure 1B.**

**
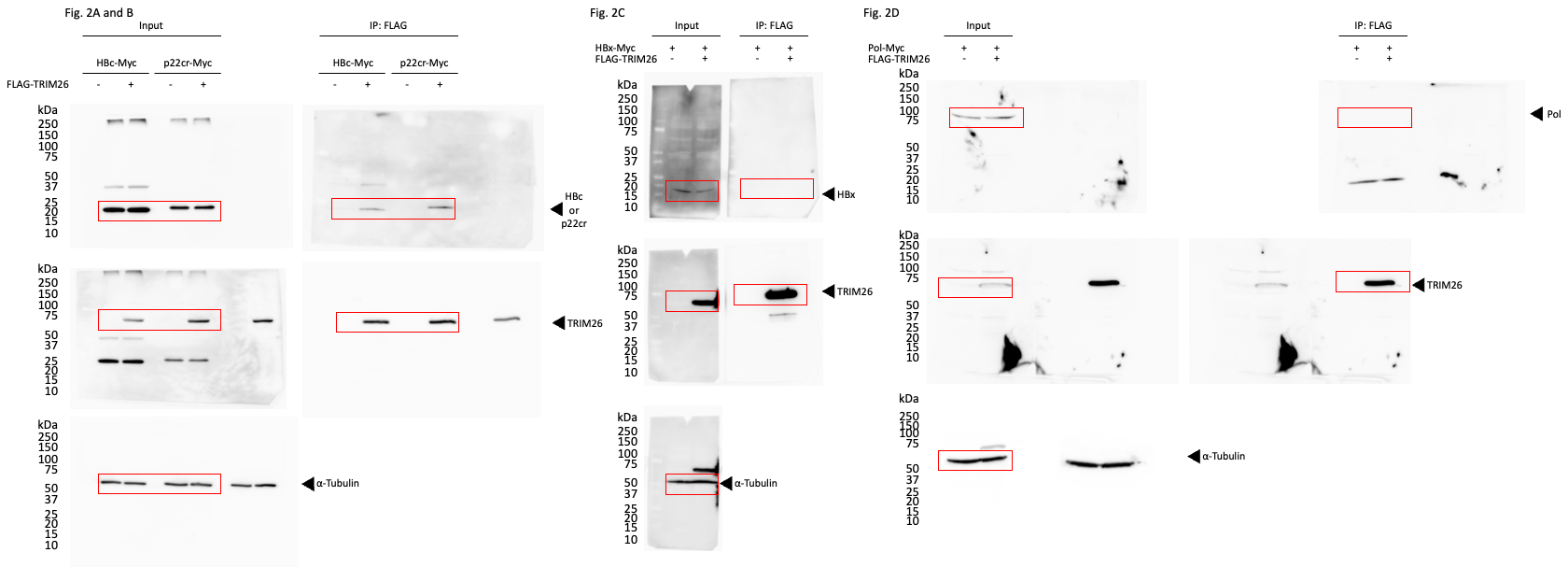
**

**Figure S7. Full-length blots for images of Figure 2.**

**
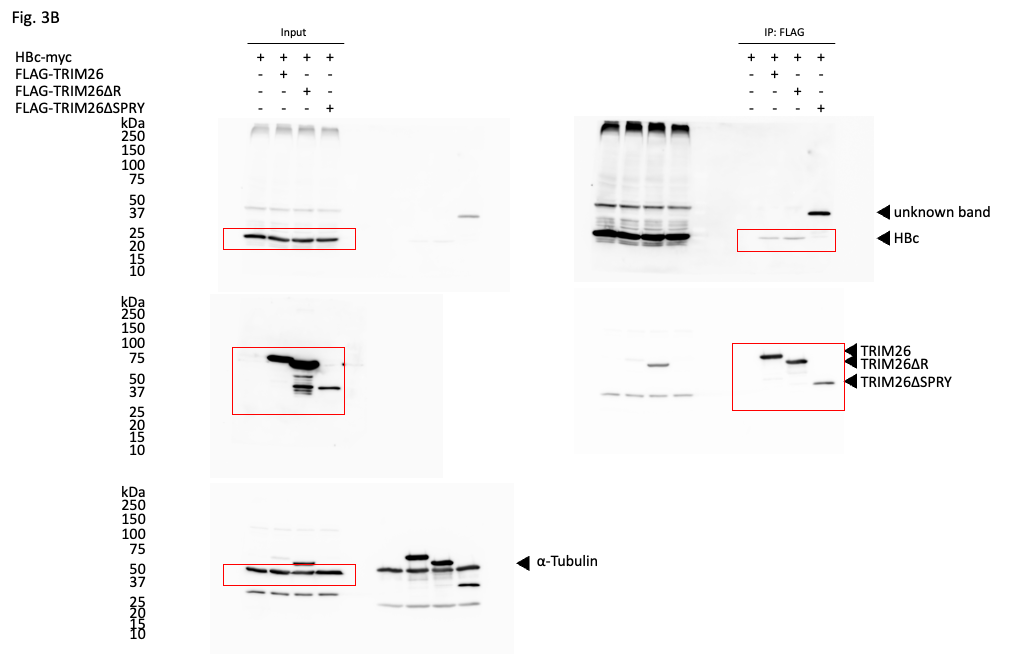
**

**Figure S8. Full-length blots for images of Figure 3.**

**
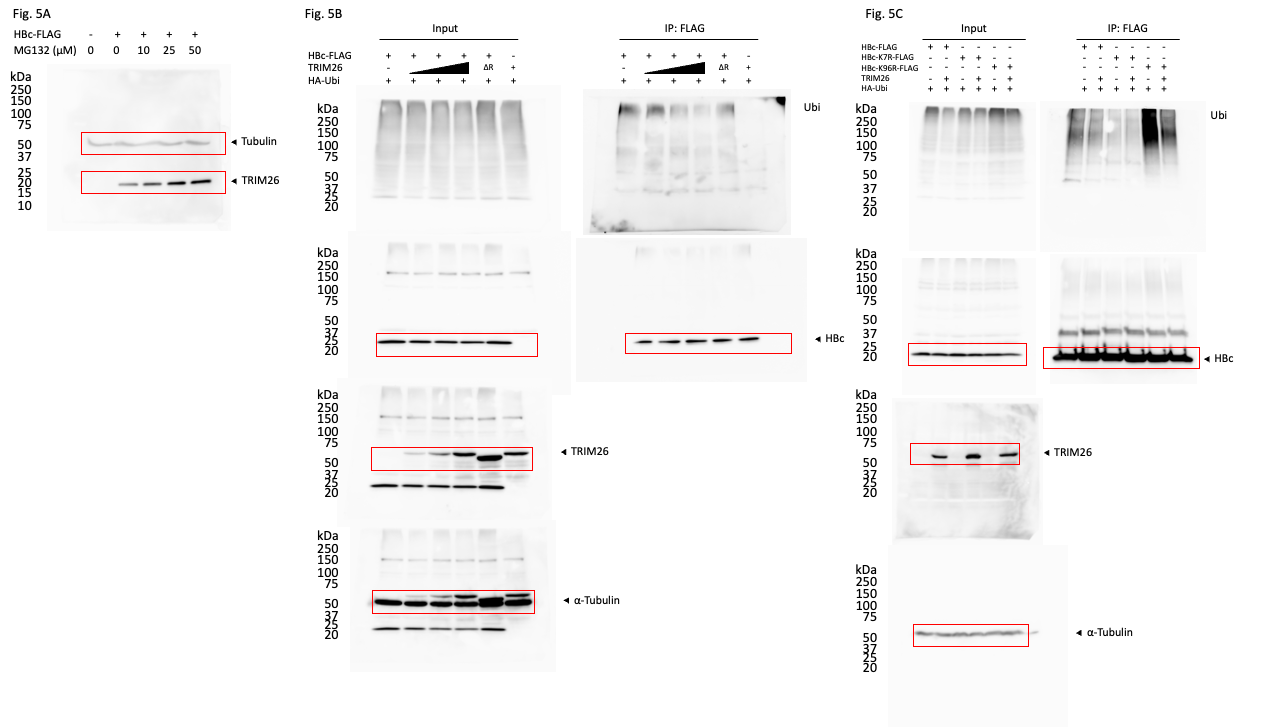
**

**Figure S9. Full-length blots for images of Figure 5.**

**
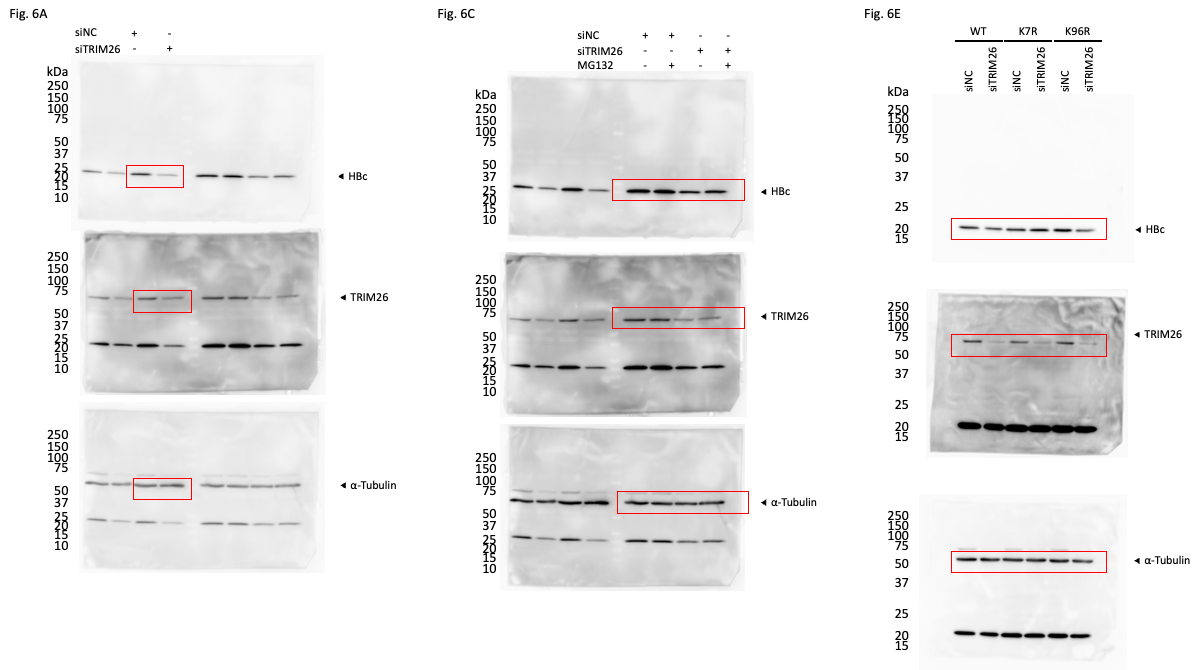
**

**Figure S10. Full-length blots for images of Figure 6.**

**
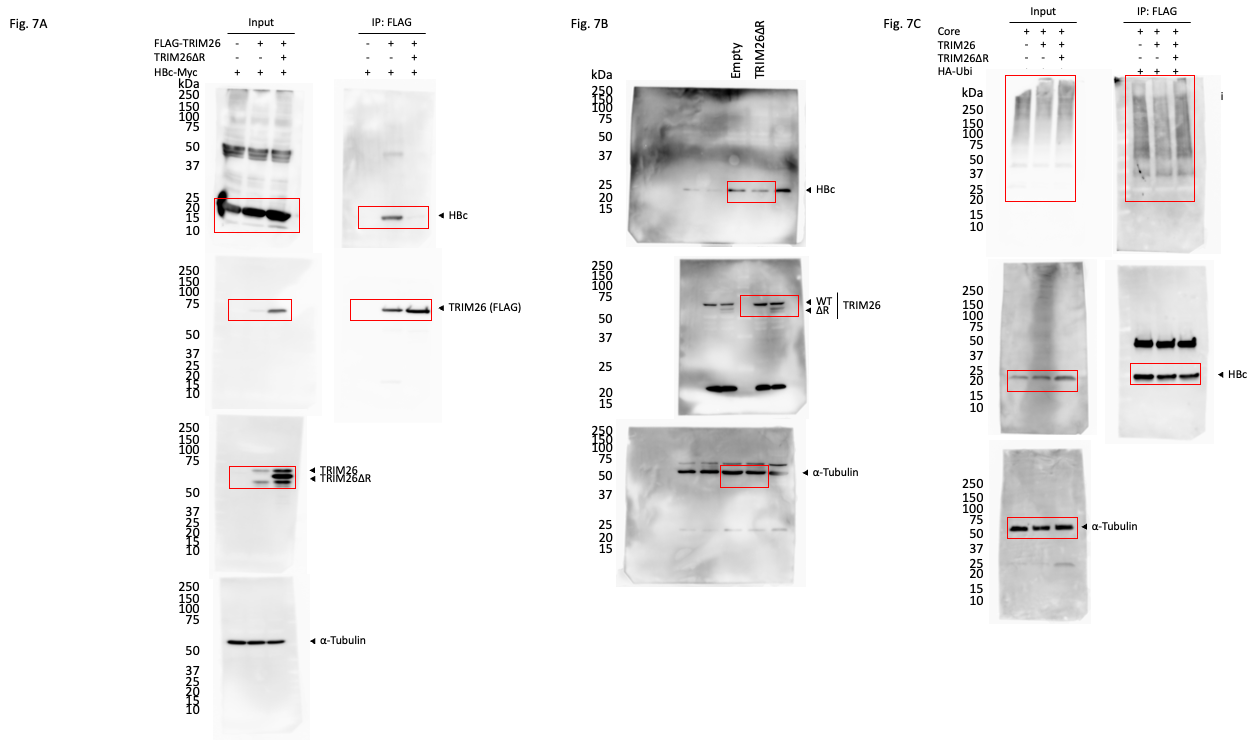
**

**Figure S11. Full-length blots for images of Figure 7.**

**
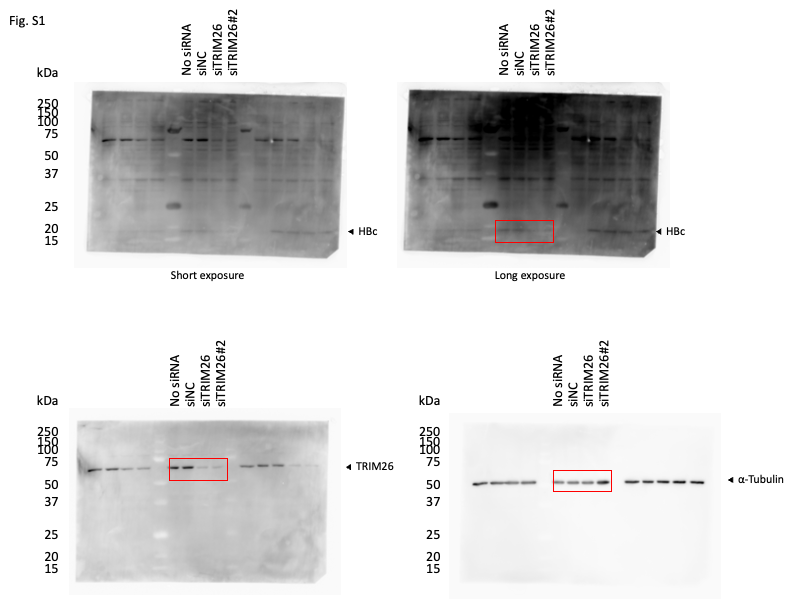
**

**Figure S12. Full-length blots for images of Figure S1.**

**
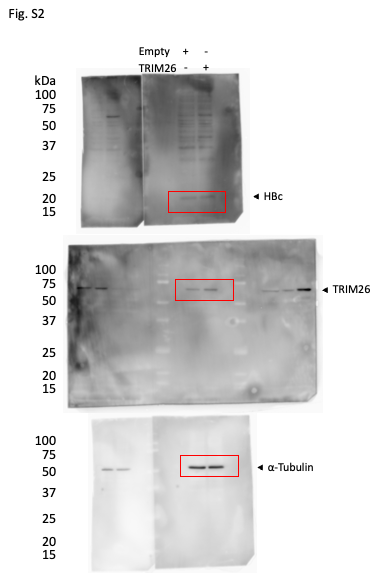
**

**Figure S13. Full-length blots for images of Figure S2.**

**
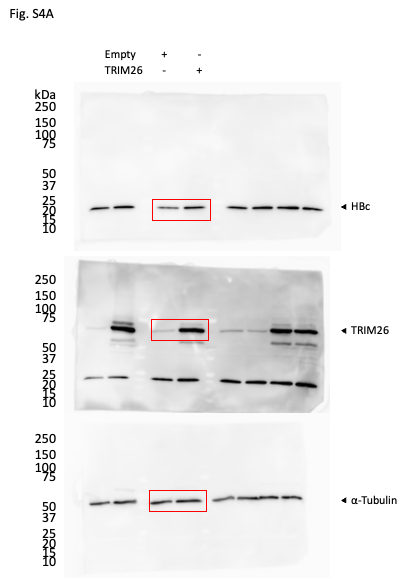
**

**Figure S14. Full-length blots for images of Figure S4A.**

**
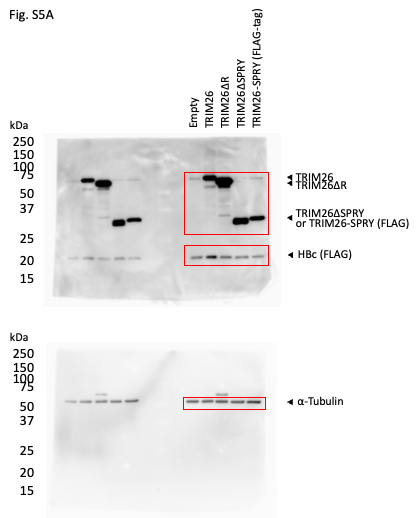
**

**Figure S15. Full-length blots for images of Figure S5A.**
